# Autointoxication

**Published:** 1904-12

**Authors:** 


### AUTOINTOXICATION.*

One of the most frequent conditons met with in considering the etiological factors in the production of disease is that of autointoxication. During the early period of the germ theory of disease the germs themselves were regarded as the maleficent agents in the production of disease processes. Later on it was contended by many eminent authorities that it was not the germs themselves, but the toxic materials or toxins which they generated that caused the trouble. And within still more recent years the theory that the body without the introduction of any external disease-producing germ or toxic materials can, by a retrograde metamorphosis of its own tissue, produce virulent poisons which may and frequently do lead to serious derangements and even cause death. We may therefor, with Weber, define autointoxication as a poisoning of the system by the products of tissue metamorphosis; which products may be normal, but do harm by excessive accumulation, or they may be pathological, either not at all or in minute quantities only, present in the healthy body. This is well illustrated in the weariness often following excessive muscular action often

*Read by C. W. Pfeiffer, M.D., before the Western District Medical Society at Quincy, Ill., October 28, 1904.

assuming the form of distress, and if the effort be continued the distress may become so great as to cause complete exhaustion that even death may result. In excessive work of whatever kind it may be, in order to accomplish this work the demand upon the blood for oxygen is increased. There are numerous things besides carbonic acid gas which are swept into the blood as a result of the activities of the body. In other words, the product of work in the human body is poison which must be eliminated through the excretory organs.

Fatigue fever, as described by Vaughn and others, occurred in this manner: Fever, as generally considered, is an increase of heat production beyond that of heat dissipation, an agent arising from within or without deranging the harmony of the thermotoxic, thermogenetic and thermolytic apparatus. Once established the fever continues not from excessive production of heat, but from the altered relation between heat production and heat dissipation.

Fever is not a phenomena of excessive oxidation of the constituents of the body.

Heat production may be subnormal, yet the temperature of the body may be very high. The equilibrium between heat production and heat dissipation is maintained by the thermotoxic centers of the brain keeping the bodily temperature at 98.4. Another very important factor in heat regulation are the peripheral endings of the sensory nerves influencing the thermolytic centers. Nerve shock or allied conditions giving rise to disturbance of the thermic balance is often productive of the autotoxemia underlying saburral fever which presents about the following clinical picture: It begins with loss of appetite, tongue is large, white and dirty. Clammy mouth; fetid breath; habitual constipation. The patient is languid, feeble, indisposed for work. This condition is often supervened by paroxyms of chills and followed with sweats. Under the influence of overwork, emotion, exposure to cold, etc., arrest of digestion, nausea, vomiting, headache, chills and fever occur. In children vertigo and cónvulsions often usher in this disease as is also the case with neurotics. In view of these facts as to the formation of toxic materials by strong muscular activity, I have been lead to believe that many cases of mild poisoning following parturition in which every possible precaution has been taken to avoid external infection, are caused by the long continued uterine action, which if intense and tonic, in all probability generate toxic materials more rapidly than they can be eliminated, especially if the uterus has been stimulated by the administration of ergot during labor. It is therefore of the utmost importance to keep all the excretory functions of the pregnant and parturient woman in first-class working order. Furthermore experience in a limited number of cases has convinced me that fractional doses of calomel with potassium acetate and spirits of nitrous ether with a little tincture of aconite if the latter is indicated, given for a day or two, will do more toward arousing the excretory organs and poison-destroying powers of the system and relieve the threatening puerperal infection more effectively than any other kind of medication. In the preface to Oliver's translation of Bouchard's work, he says as follows: Bouchard in his autointoxication clearly indicates to us that man is constantly standing on the brink of a precipice; he is continually on the threshold of disease. Every moment of his life he runs the risk of being overpowered by poison generated within his system. Self-poisoning is only prevented by the activity of the excretory organs, chiefly the kidneys and by the watchfulness of the liver, which acts the part of a sentinel to the materials brought to it by the portal vein from the alimentary canal.

The place held by autotoxemia in the production of mental diseases is second only to heredity. Peterson in describing the causes of insanity uses two terms, heredity and strain; heredity, which renders the nervous organization unstable, the strain, which causes the unstable nerve-centers to collapse. It has long been known that the fluids in the body of the insane undergo modification. Recent investigation has shown that the urine in the insane is much less toxic than in the normal condition; while the lethal action of this fluid is increased in melancholia. Maniacal urine gives rise to excitement and convulsions when injected into an animal, while the injection of urine taken from a case of melancholia is followed by a depression of spirits, restlessness and stupor, a proof that autointoxication is the cause and not the effect of a mental condition.

Bouchard has also shown that urine taken from a healthy person if injected into animals produces toxic symptoms, and when used in sufficient quantities will cause death. From a careful study of a large number of experiments conducted on animals he arrives at the conclusion that the urine contains substances which produce the following effects:

1. A diuretic substance.
2. A narcotic substance.
3. A convulsive substance.
4. A sialogenous substance.
5. A substance producing contraction of pupil.
6. A heat-reducing substance.

I believe that every observing physician could bear witness to the fact that many cases of depression of spirits, mental hebetude and general pessimism are due not to some incipient serious organic disease, as the patent medicine manufacturer or the blatant advertising charlatan would have these sufferers believe, but are merely the result of the accumulation of retrograde toxic products by the normal activities of their bodies, which instead of being promptly eliminated, accumulate in the blood and poison the nerve-centers, set up disorders of the digestive organs, overtax the liver and lead to almost innumerable functional derangements. It is now well understood that not only are diseases directly connected with the digestive and urinary organs caused by autointoxication, but likewise disorders of the distant and special organs are due to the same cause, chief among which are amblyopia and that form of optic atrophy which Uthoff placed among the unknown causes.

While not relaxing our care and vigilance to prevent the introduction of external infective materials yet I believe the facts to which I have called attention should serve to broaden our views of the causes of disease and increase our vigilance in preventing them and enlarge our power of combating them when existent.

While such a course should not relax our duty in searching for the specific bacilli and their toxins which cause many diseases, it should at the same time enlarge our mental horizon and impress upon our minds the fact that our whole duty consists not only in identifying the germ and discovering a germicide to destroy it, but the patient should receive our most serious and earnest consideration in our endeavor to secure elimination of the peccant materials by correcting and sustaining the proper function of the excretory organs, thereby increasing the resistance of cells and tissues in their battle against their destroyers. The therapeutics of autotoxemia will depend largely upon the type. In cases of irritable weakness a change of environment is desired for the most rapid and permanent recovery. Much may be attained by regulation of diet, use of the mineral waters, regular and moderate exercise as well as massage and hydrotherapy. In this trouble the great need of the system is water, and it should be amply supplied, for which the ordinary water will suffice if mineral waters for some reason can not be procured. Large quantities of milk, or better still, buttermilk will act admirably in a number of cases. Purgatives require very careful adjustment.—*The Medical Fortnightly.*

